# Risks and benefits of bisphosphonates

**DOI:** 10.1038/sj.bjc.6604382

**Published:** 2008-05-27

**Authors:** R E Coleman

**Affiliations:** 1 Academic Unit of Clinical Oncology, Weston Park Hospital, University of Sheffield, Sheffield S10 2SJ, UK

**Keywords:** bisphosphonates, bone metastases, treatment induced bone loss

## Abstract

Bone is the most common site for metastasis in cancer and is of particular clinical importance in breast and prostate cancers due to the prevalence of these diseases. Bone metastases result in considerable morbidity and complex demands on health care resources, affecting quality of life and independence over years rather than months. The bisphosphonates have been shown to reduce skeletal morbidity in multiple myeloma as well as a wide range of solid tumours affecting bone by 30–50%. Quite appropriately, these agents are increasingly used alongside anticancer treatments to prevent skeletal complications and relieve bone pain.

The use of bisphosphonates in early cancer is also increasingly important to prevent the adverse effects of cancer treatments on bone health. These include ovarian suppression and the use of aromatase inhibitors in breast cancer patients and androgen deprivation therapy in those with prostate cancer. Bisphosphonate strategies, similar to those used to treat postmenopausal osteoporosis, have suggested that bisphosphonates are a safe and effective treatment for the prevention of treatment-induced bone loss.

When compared to other cancer therapies, the frequency and severity of adverse events related to bisphosphonate therapy are generally mild and infrequent; thus, the benefits of treatment with any bisphosphonate almost always outweigh the risks. However, renal dysfunction may occasionally occur and over recent years, a new entity, bisphosphonate-associated osteonecrosis of the jaw (ONJ), has been described. The incidence, clinical importance and prevention strategies to minimise the impact of this problem on patients requiring bisphosphonates is discussed.

Bone is a common site for metastasis in patients with solid tumours arising from the breast, prostate, lung, thyroid and kidney (Brown *et al*, 2005). Approximately 70% of patients with advanced prostate cancer or breast cancer and up to 40% of patients with other advanced solid tumours will develop bone metastases. Additionally, in more than 50% of men with advanced prostate cancer and around 20% of women with advanced breast cancer, metastatic disease appears clinically confined to the skeleton.

Bone metastases are typically referred to as osteoblastic, osteolytic or mixed, based on the radiographic appearance of the lesions. However, osteoblastic and osteolytic bone lesions represent two extremes of a spectrum and osteoclast activity is increased in most bone metastases, including the typical osteoblastic metastases from prostate cancer (Coleman, 2006). Pathological activation of osteoclasts appears to play a central role in most disease-related skeletal complications and is, thus, a rational therapeutic target and the basis for the use of bisphosphonates across the range of cancers affecting bone (Dunstan *et al*, 2007).

Metastatic bone disease disrupts the normal homeostasis of bone, a dynamic process that involves the coupled and balanced osteoclast-mediated osteolysis and osteogenesis by osteoblasts (Coleman, 2006). The resulting increased and unbalanced bone metabolism leads to a loss of bone integrity, which may result in skeletal morbidity. This includes bone pain, pathological fractures, a need for orthopaedic surgery to prevent or repair major structural damage, spinal cord and nerve root compression and hypercalcaemia of malignancy. Bone pain is the most prevalent of all cancer-associated types of pain, and may be severe, necessitating opiate analgesics and palliative radiation therapy and often accompanied by a substantial decline in patient-reported quality of life. Despite the many advances in the treatment of advanced cancer over recent decades, skeletal morbidity remains a major clinical problem with annual fracture rates of 20–40% and the occurrence of a significant skeletal complication every 3–6 months in the absence of bone-targeted therapies, such as bisphosphonates (Brown *et al*, 2005).

In addition to the effects of spread of cancer to bone, there are also important effects of cancer treatments on bone health. This is largely mediated through endocrine effects of treatment, including ovarian suppression, androgen deprivation therapy and the use of aromatase inhibitors that block oestrogen synthesis in postmenopausal women. With the improved survival that has emerged over the past 30 years through improved diagnostic and therapeutic interventions, the long-term effects of treatment on the skeleton have become an important clinical problem and a specific rationale for the use of bone-specific treatments (Lester *et al*, 2005).

Over the past two decades, the bisphosphonates have become established as an important component of treatment in metastatic disease and the management of osteoporosis. All bisphosphonates are pyrophosphate analogues, characterized by a P-C-P containing central structure rather than the P-O-P of pyrophosphate, and a variable side chain (Roelofs *et al*, 2006). The P-C-P backbone renders bisphosphonates resistant to phosphatase activity and promotes their binding to the mineralised bone matrix.

After intravenous administration of a bisphosphonate, approximately 50–75% of the injected dose binds avidly to exposed bone mineral, where it is internalised by the osteoclast during bone esorption and the remainder is excreted by the kidney. All bisphosphonates suffer from poor bioavailability, when given by mouth (<5%), and must be taken on an empty stomach, as they bind to calcium in the diet. Once a bisphosphonate is taken up by an osteoclast, it causes the disruption of a number of biochemical processes involved in osteoclast function, typically resulting in apoptosis of the cell.

The molecular mechanism of action is now established, with nitrogen-containing bisphosphonates inhibiting farnesyl diphosphatase and other enzymes of the mevalonate pathway (Roelofs *et al*, 2006). Non-nitrogen-containing bisphosphonates, such as clodronate, have a different mode of action and induce osteoclast apoptosis through the generation of cytotoxic ATP analogues (Roelofs *et al*, 2006). Recent studies also suggest that bisphosphonates have direct effects on tumour cells (Clezardin 2005) and may enhance the antitumour activities of cytotoxic agents (Neville-Webbe *et al*, 2005).

## TREATMENT BENEFITS

### Bisphosphonates to prevent skeletal morbidity and relief of bone pain

Radiotherapy is the treatment of choice for localised bone pain from metastasis, but the bisphosphonates provide an additional treatment approach, especially for patients with poorly localised bone pain or recurrence of bone pain in previously irradiated sites. Approximately, one half of patients demonstrate significant improvement in pain, an effect that appears to be independent of the nature of the underlying tumour or radiographic appearance of the metastases (Wong and Wiffen, 2002).

Additionally, based on the results of large randomised controlled trials, conducted in the late 1990s, the bisphosphonates have become the standard of care for the treatment and prevention of skeletal complications associated with bone metastases in patients with breast cancer and multiple myeloma (Coleman, 2004). More recently, as summarised in Table 1, they have also demonstrated benefits in patients with bone metastases secondary to other cancers, including prostate cancer (Saad *et al*, 2004), lung cancer (Rosen *et al*, 2004) and a broad range of other solid tumours (Rosen *et al*, 2004). This prophylactic use of bisphosphonates in managing bone metastases has had a profound beneficial effect on the frequency and severity of skeletal morbidity. With the use of modern potent agents, the proportion of patients suffering from skeletal-related events (SREs) has been reduced by 30–50% and this reduction has resulted in significant and clinically important benefits in quality of life and preserved function (Coleman, 2004).

Outside the United States, the choice of available agents for use in oncology is considerable and includes both oral and intravenous formulations of ibandronate and clodronate, and intravenous zoledronic acid and pamidronate. The optimal type and route of administration for a bisphosphonate to prevent skeletal morbidity remains uncertain due to the lack of head-to-head comparisons between the intravenous and oral agents. Zoledronic acid, however, has proven effective across the range of solid tumours, whereas the efficacy benefits of the other agents are restricted to breast cancer and myeloma. Typically, in metastatic bone disease, intravenous formulations are administered every 3–4 weeks and oral agents given on a daily basis. With the latter, strategy compliance may be a significant problem (Cramer *et al*, 2007).

Cancer treatment-induced bone loss may also be an indication for bisphosphonate use, especially if the subject has low bone mineral density or risk factors for the occurrence of a low trauma fracture (Lindsay *et al*, 1997). In this setting, the disturbance of bone turnover is more similar to that seen in postmenopausal bone loss and doses, and schedules of bisphosphonate treatment that are similar to those used in osteoporosis are sufficient. Intravenous zoledronic acid every 6 months (Gnant *et al*, 2007), monthly oral ibandronate (Lester *et al*, 2007) and weekly oral risedronate (Van Poznak *et al*, 2007) have all been shown to prevent bone loss associated with the use of aromatase inhibitors for postmenopausal breast cancer. In the context of androgen-deprivation therapy, zoledronic acid every 3–12 months is able to prevent the bone loss associated with therapy (Smith *et al*, 2003; Michaelson *et al*, 2007).

## TREATMENT RISKS

When compared to other cancer therapies, the frequency and severity of adverse events related to bisphosphonate therapy are generally mild and infrequent; thus, the benefits of treatment with any bisphosphonate almost always outweigh the risks. The side-effect profile is mostly influenced by the administration route. With intravenous amino-bisphosphonates, around 15–30% of patients will experience an acute phase reaction probably related to the stimulation of *γδ*T cells and characterized by transient fever and muscle and joint aches but this usually only follows the first infusion and is largely irrelevant thereafter. Some data suggest that the incidence of the acute phase response is less common in immunocompromised advanced cancer patients than in healthy subjects or in those without metastases.

### Renal complications

Renal abnormalities have been described with intravenous agents, particularly when they are given at high doses (above standard) or as a rapid infusion. However, at the recommended dose and schedule of any bisphosphonate, renal toxicity is unusual, usually predictable and reversible, and serious bisphosphonate-induced renal complications (including renal failure) are rare (<0.5%) (Guarneri *et al*, 2005). Because renal dysfunction is infrequent, none of the placebo-controlled trials with ibandronate or zoledronic acid (when given at the currently recommended dose and schedule) showed any statistically significant differences between active therapy and placebo in creatinine levels with time. However, idiosyncratic renal abnormalities undoubtedly do occur. With pamidronate a focal glomerulosclerosis associated with nephrotic syndrome is recognised, whereas with the use of zoledronic acid, the renal abnormalities relate to tubular damage (Guarneri *et al*, 2005).

There are suggestions that ibandronate is less likely than zoledronic acid or pamidronate to influence renal function. This has been attributed to the higher protein binding and prolonged elimination half-life of ibandronate. There are undoubtedly more case reports of renal dysfunction with zoledronic acid because the drug is so widely used, but whether there truly is a renal safety advantage to ibandronate remains uncertain. In the absence of appropriately powered randomised comparative trials, it is not possible to conclude on whether ibandronate truly has tolerability advantages over the other agents.

### Osteonecrosis of the jaw

In recent years, ONJ has become one of the most discussed adverse events in advanced malignancy; partially stimulated by the publication of photographs of extreme cases that often were worsened by inappropriate surgery (Marx, 2003; Ruggiero, 2004). In addition, these initial case series neither gave estimation of the incidence of the problem nor indicated that most cases may be managed conservatively using oral rinses and antibiotics.

Since the original reports of ONJ associated with the use of bisphosphonates were produced 5 years ago, over 1000 other cases have come to the attention of regulatory authorities around the world and an ever-increasing number of case series, retrospective cohort studies and insurance claim reviews have been published (Ruggiero *et al*, 2006; Wilkinson *et al*, 2007). These studies have not used a consistent definition for ONJ, although this has now been defined as an area of exposed bone in the maxillofacial region that did not heal within 8 weeks after identification by a health care provider, in a patient who was receiving or had been exposed to a bisphosphonate and had not had radiation therapy to the craniofacial region (Khosla *et al*, 2007). Additionally, the methods of data collection have varied; in some cases, they have used inherently biased internet surveys or retrospective chart reviews. The frequency of ONJ suggested by these exploratory reports has varied considerably between <1 and >10% (Wilkinson *et al*, 2007). In the largest chart review from the MD Anderson Cancer Centre, 4000 patients treated with bisphosphonates were reviewed (Hoff *et al*, 2006). The overall incidence of ONJ, using a definition of exposed bone for at least 3 months, was 0.83% with an approximate time-dependent rate of 1% per year on treatment. This could be an underestimate due to the inclusion of patients who received only a single dose for hypercalcaemia. However, these patients were treated before careful pretreatment, dental assessment and avoidance of invasive dental procedures were instituted, strategies that have almost certainly reduced the frequency of ONJ (American Dental Association Council on Scientific Affairs, 2006).

The pathogenesis of ONJ remains obscure. Local oversuppression of bone turnover in the jaw, and inhibition of angiogenesis by high doses of potent amino-bisphosphonates complicating trauma and infection of the jaw are the most likely contributing factors, but a definite causal relationship between bisphosphonate use and ONJ remains uncertain and complicated by factors that may increase the risk of developing the condition, such as the concomitant use of cancer treatments and/or corticosteroids, requirements for dental surgery and comorbidities, such as anaemia and diabetes (Hoff *et al*, 2006). Seventy-five percent occur following a dental extraction or jaw surgery. Histopathologically exposed necrotic bone, usually with an inflammatory inflitrate and evidence of infection with a mixed bacterial growth often containing actinomyces species, is observed.

Information on ONJ from randomised clinical trials is very limited. A retrospective analysis of over 3000 patients included in the zoledronic acid development programme identified a maximum of six patients who might, in hindsight, have experienced ONJ, although some of these individuals may have had osteomyelitis or other dental conditions (Novartis, 2005). In a recent randomised trial of IV zoledronic acid, given to treat osteoporosis, there were two possible cases of ONJ, one in the placebo arm and one in the group receiving active treatment (Black *et al*, 2007).

Prospective research is required to determine the causes of ONJ and the frequency of the condition using a consistent definition in cancer patients treated with and without bisphosphonates. The relationships, if any, with the type, dose and duration of bisphosphonate treatment need to be defined, as do the strategies for prevention, early diagnosis and subsequent treatment. In the meantime, patients should have regular inspection of the mouth, undergo dental checkups every 6–12 months and avoid invasive dental surgery unless no alternative is available (Hoff *et al*, 2006; Wilkinson *et al*, 2007).

### Optimising the risk/benefit ratio

Consensus guidance recommendations indicate that all patients with multiple myeloma (American Society of Clinical Oncology, 2007) and radiologically confirmed bone metastases from breast cancer (Hillner *et al*, 2003) should receive bisphosphonates from the time of diagnosis of bone metastasis and continued indefinitely.

The development of an SRE is not a specific sign of treatment failure or a signal to stop treatment; evidence is now available to confirm that bisphosphonates delay second and subsequent complications, not just the first event. However, a recent report suggested that switching to a more potent agent (zoledronic acid) may be an appropriate option in breast cancer patients in whom pamidronate or clodronate therapy proves ‘unsatisfactory’ (Clemons *et al*, 2006).

Evidence-based criteria are needed to determine when in the course of metastatic bone disease the bisphosphonates should be started and stopped. Because of the logistics and cost of delivering monthly intravenous infusions for all patients with metastatic bone disease, empirical recommendations on who should receive treatment that take into account the underlying disease type and extent, the life expectancy of the patient, the probability of the patient experiencing a SRE and the ease with which the patient can attend for treatment (or be treated by a domiciliary service) appear appropriate (Dunstan *et al*, 2007).

Despite the obvious clinical benets of bisphosphonates, only a proportion of skeletal events are prevented. Additionally, some patients do not experience a skeletal event despite the presence of bone metastases. Bisphosphonates are a relatively costly additional intervention in cancer care that is now potentially applicable to a very large proportion of patients with advanced malignancy. Although bisphosphonate use in some disease settings may be associated with cost savings (Botteman *et al*, 2006), the cost effectiveness of routine long-term treatment across all disease types has been questioned (Reed *et al*, 2004; McKeage and Plosker, 2008), and this, coupled with the small but finite risk of toxicity, suggests that a more selective use of bisphosphonates would be appropriate.

Biochemical markers of bone metabolism provide valuable prognostic information in patients with metastatic bone disease and monitoring of bone resorption markers may be useful to identify patients at high risk of skeletal complications and guide the frequency of treatment (Brown *et al*, 2005; Coleman *et al*, 2005). In an analysis of patients with bone metastases from breast cancer, prostate cancer, lung cancer or other solid tumours, or bone lesions from multiple myeloma that elevated on treatment levels of N-telopeptide of type 1 collagen (NTX) were associated with a significant 2-fold increased risk of disease progression and risk of skeletal complications (*P* <0.001 for all). In addition, elevated NTX levels were associated with a significant two- to threefold increased risk of experiencing a first SRE on study in patients with breast cancer, prostate cancer or multiple myeloma (*P*⩽0.008). Furthermore, elevated NTX levels were associated with a significant four- to sixfold increased risk of dying on study in all solid tumour patients (*P*<0.001 for all) (Coleman *et al*, 2005).

A more cost-effective use of bisphosphonates, particularly in patients with additional extensive visceral metastases or solid tumours associated with a short-life expectancy, might be to reserve them until patients have raised NTX levels and to adjust the frequency of treatment to maintain levels of bone resorption within the normal range, increasing the interval between treatments when the disease is in remission and the rate of bone resorption is normal. Bone-marker-directed therapy is under evaluation in randomised clinical trials and the value of maintenance therapy after 1 to 2 years of treatment may become clear from the ongoing Optimize trial in the United States.

## CONCLUSION

The use of bisphosphonates in oncology has had a profound beneficial effect on the management of metastatic bone disease and the prevention of treatment-induced bone loss. Their use should be considered in all patients with bone metastases, especially those with symptoms and without immediately life-threatening extraskeletal disease. Guidelines for the use of the agents in preventing treatment-induced bone loss are evolving and trials investigating their potential role in the adjuvant setting to prevent metastasis are ongoing (Table 2). As a class, the agents are well tolerated. Occasional serious toxicities in terms of renal impairment and ONJ can be largely avoided through adhering to the recommended dose and infusion times and good preventative dental care, respectively.

## Figures and Tables

**Table 1:** Effects of bisphosphonates on skeletal morbidity: results of randomized placebo-controlled trials

**Agent and route**	***N***	**Results**	**Investigator**
*Breast cancer*			
Clodronate 1600 mg po *vs* placebo	173	Reduced SMR– 305 *vs* 219 events/100 woman years (*P*=<0.001)	Paterson
Pamidronate 45 mg IV *vs* control	295	Increased time to bone progression–168 *vs* 249 days (*P*=0.02)	Conte
		Delay in rst SRE–7.0 months *vs* 13.1 (*P*=0.0005)	
Pamidronate 90 mg IV *vs* placebo	382	Reduced proportion experiencing SRE–65% *vs* 46% (*P*=<0.001)	Hortobagyi
Pamidronate 60 mg IV *vs* control	401	Median time to skeletal progression –9 *vs* 14 months (*P*=<0.01)	Hultborn
Pamidronate 90 mg IV *vs* placebo	374	Reduced proportion experiencing SRE–67 *vs* 56% (*P*=0.027)	Theriault
		Delay in rst SRE 6.9 months *vs* 10.4 (*P*=0.049)	
Ibandronate 2/6 mg IV *vs* placebo	467	Reduced SMR with 6 mg dose, 2 mg ineffective–SMR 2.18 *vs* 1.61 (*P*=0.03)	Body
Zoledronic acid 4 mg iv *vs* placebo	227	Reduced proportion experiencing SRE–50 *vs* 30% (*P*=0.003)	Kohno
		Reduced SMR by 43% (*P*=0.016)	
			
*Multiple myeloma*			
Clodronate 1600 mg po *vs* placebo	350	Improved 2-year progression-free survival–24 *vs* 12% (*P*<0.05)	Lahtinen
Pamidronate 90 mg IV *vs* placebo	392	Reduced proportion experiencing SRE–24 *vs* 41% (*P*<0.001)	Berenson
Clodronate 1600 mg po *vs* placebo	614	Less skeletal morbidity and pain on progression	McCloskey
			
*Prostate cancer*			
Clodronate (4 × 520) mg oral *vs* placebo	311	Reduction in number of SREs *vs* placebo not significant –(49 *vs* 41%, *P*=NS)	Dearnaley
Pamidronate 90 mg iv *vs* placebo	378	Number of SREs equal in pamidronate and placebo-arms, *P*=1.0	Small
Zoledronic acid 4/8 mg *vs* placebo	643	25% reduction in proportion of patients experiencing at least one SRE–(*P*=0.021)	Saad
			
*Other tumour types*			
Zoledronic acid 4/8 mg *vs* placebo	773	Significant delay to time of rst SRE (*P*=0.023)	Rosen
		Reduction in proportion of patients with SRE–(47 *vs* 38%, *P*=0.039)	

SRE, skeletal-related event.

**Table 2:** Clinical trials of bisphosphonates to prevent metastasis

**Agent and trial descriptor**	**Setting**	**Treatment and duration**	**Study size and status**	
*Clodronate (oral)*				
NSABP-B34	Stage I–III breast cancer	3 years clodronate *vs* placebo	3400	Accrual complete
				
*Zoledronic acid (iv)*				
AZURE	Stage II/III breast cancer	5 years zoledronic acid *vs* control	3360	Accrual complete
Z-FAST/ZO-FAST/EZO-FAST	Stage I–III breast cancer	Immediate *vs* delayed zoledronic acid	2193	Accrual complete
ABCSG XII	Stage I-II breast cancer	6 monthly zoledronic acid *vs* control	1800	Accrual complete
SUCCESS	Stage I–III breast cancer	3 *vs* 5 years zoledronic acid	3754	Acccrual ongoing
ZEUS	High-risk prostate cancer	3 years zoledronic acid *vs* control	1433	Accrual complete
2419	Stage IIIA/IIIB NSCLC	2 years zoledronic acid *vs* control	446	Accrual ongoing
				
*Ibandronate (oral)*				
GAIN	Stage II/III breast cancer	2 years ibandronate *vs* control	>3000	Accrual ongoing
				
*Others*				
S0307/Intergroup	Stage I–III breast cancer	3 years clodronate *vs* zoledronic acid *vs* ibandronate	4500	Accrual ongoing
